# Orientation tuning of binocular summation: a comparison of colour to achromatic contrast

**DOI:** 10.1038/srep25692

**Published:** 2016-05-11

**Authors:** Mina Gheiratmand, Avital S. Cherniawsky, Kathy T. Mullen

**Affiliations:** 1 McGill Vision Research, Department of Ophthalmology, McGill University, Montreal, Quebec H3G 1A4, Canada

## Abstract

A key function of the primary visual cortex is to combine the input from the two eyes into a unified binocular percept. At low, near threshold, contrasts a process of summation occurs if the visual inputs from the two eyes are similar. Here we measure the orientation tuning of binocular summation for chromatic and equivalent achromatic contrast. We derive estimates of orientation tuning by measuring binocular summation as a function of the orientation difference between two sinusoidal gratings presented dichoptically to different eyes. We then use a model to estimate the orientation bandwidth of the neural detectors underlying the binocular combination. We find that orientation bandwidths are similar for chromatic and achromatic stimuli at both low (0.375 c/deg) and mid (1.5 c/deg) spatial frequencies, with an overall average of 29 ± 3 degs (HWHH, s.e.m). This effect occurs despite the overall greater binocular summation found for the low spatial frequency chromatic stimuli. These results suggest that similar, oriented processes underlie both chromatic and achromatic binocular contrast combination. The non-oriented detection process found in colour vision at low spatial frequencies under monocular viewing is not evident at the binocular combination stage.

A key function of the primary visual cortex in mammals is to combine the information from the two eyes into unified binocular responses occurring at the level of a single cell. Since the earliest neurophysiological experiments on V1, it has been known that the majority of neurons in the upper layers of the primary visual cortex are binocular, responding to a stimulus presented to either eye, with overall an approximately normal distribution of the relative weights of the responses from the two eyes (ocular dominance)^1^. The receptive field properties for the neural responses are similar between the two eyes in terms of their spatial frequency and orientation tuning^2^,^3^, ensuring that binocular combination only occurs when similar stimuli are presented to the two eyes. These properties have largely been investigated using achromatic contrast, however, relatively little is known about the role of colour contrast in neural binocular combination. Experiments using colour contrast in primate V1^4^ have reported that neurons with high colour sensitivity are also binocular, and are found in similar proportions to binocular, achromatically-driven neurons. Interestingly, Peirce *et al*.^4^ reported a sub population of strongly colour sensitive neurons that have closely matched colour preferences between the two eyes, but lack spatial frequency and orientation tuning. Presumably, these are formed from monocular, non-oriented units that lack a strong center-surround organization but combine their inputs into a binocular neuron based on their strong colour similarities. There are also many neurons in V1 that are sensitive to both colour and achromatic contrast (colour-luminance neurons). This population is typically orientation and spatial frequency selective^5^,^6^, and is also binocular^4^. Thus physiological evidence points to the presence of both orientation-tuned and untuned (isotropic) binocular neurons in colour vision.

Recent psychophysical experiments using monocular viewing conditions have found evidence for two types of colour responses based on their orientation and spatial frequency tuning. Using a method of subthreshold summation, Gheiratmand *et al*.^7^ and Gheiratmand & Mullen^8^ found that, at low spatial frequencies, colour detection thresholds are determined by non-oriented or very broadly tuned responses, whereas at higher spatial frequencies the responses are orientation-tuned. These experiments, however, specifically applied to monocular viewing and so may reflect the responses of monocular mechanisms. It is unclear whether this isotropic versus orientation-tuned dichotomy remains when stimuli are presented dichoptically to different eyes, so specifically engaging binocular mechanisms in the process of summation. If binocular summation is orientation-tuned, greatest summation between the two eyes will occur when the two stimuli are of similar orientations. Alternatively, an isotropic summation process would allow binocular summation between stimuli of widely different or even orthogonal orientations. Hence determining how binocular summation depends on the orientation difference between stimuli presented to the two eyes provides a means of assessing the orientation tuning of the binocular summation process. Here we address two linked questions. First, are the previously measured isotropic colour responses strictly monocular or can they also be found for binocular responses? Second, is the orientation tuning of the binocular response broader for colour than achromatic contrast? The poorer contrast sensitivity of the stereoscopic responses of colour vision^9^ might reflect fewer or less selective binocular responses. While this selectivity has not been tested, binocular summation is actually greater for colour contrast under ideal circumstances^10^,^11^. So far, the orientation tuning of binocular summation for colour and even achromatic contrast is largely unknown.

To address these questions, we develop a psychophysical subthreshold summation method to investigate the orientation tuning of the binocular inputs in colour vision and, for comparison, in achromatic vision, across different spatial frequencies. We derive estimates of the orientation bandwidth of the binocular chromatic and achromatic responses by measuring binocular summation for stimulus detection as a function of the orientation difference (0 to 90 degs) between two sinusoidal gratings, presented dichoptically to different eyes. We use a model-based approach, taking into account the effect of stimulus bandwidth and spatial summation in order to extract the bandwidths of the underlying binocular detectors from these orientation-tuning functions. Measuring binocular summation at detection threshold is important. The subthreshold summation method has a significant advantage over other high contrast masking methods because it uses very low, near threshold contrasts and therefore avoids the inter-ocular (dichoptic) suppressive effects that typically occur when high contrast stimuli of different orientations are presented to the two eyes^12^,^13^. This allows estimates of the underlying bandwidths of the binocular summation process without influence from the broadly tuned suppressive effects that dominate measurements made using suprathreshold, high contrast masking.

## Results

### Tuning curves

**Figure 1:**
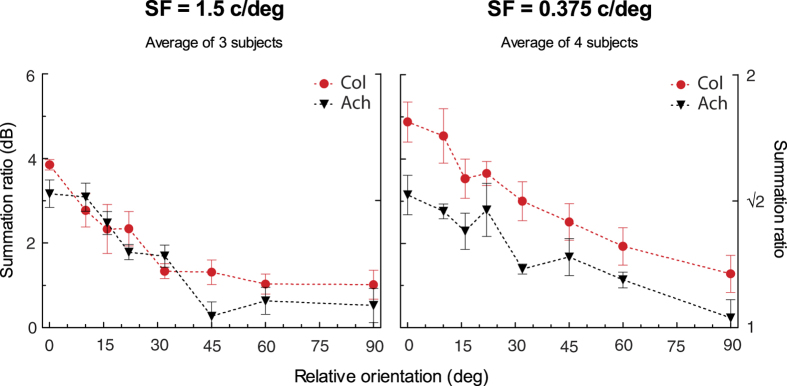
Binocular orientation tuning curves. Summation ratio in dB (left axis) or per 1 (right axis) plotted as a function of the relative orientation of the grating pairs presented dichoptically for chromatic (red circles) and achromatic (black triangles) stimuli at 1.5 c/deg (average of three subjects) and 0.375 c/deg (average of four subjects). Error bars on the averaged data show the standard error of the mean (s.e.m.).

Figure 1 shows the subject averaged binocular summation data plotted as a function of the orientation difference between the two gratings for colour and achromatic stimuli at mid (1.5 c/deg) and low (0.375 c/deg) spatial frequencies. The functions for individual subjects are shown in the Supplementary Figure S1. Binocular summation is highest when the plaid consists of co-oriented gratings and summation levels fall as orientation differences increase. For co-oriented gratings we see a significant improvement in sensitivity for binocular colour and luminance vision at levels higher than those expected from probability summation alone^10^,^14^,^15^,^16^, confirming the existence of binocular neural mechanisms in red-green colour vision. Colour vision shows greater binocular summation levels compared to luminance vision, consistent with previous findings^10^,^11^. Our results also suggest a spatial frequency dependency for colour vision’s superior binocular summation. At low spatial frequencies, for co-oriented gratings, colour contrast has a binocular summation ratio of 1.76 (±0.10, s.e.m.) compared to luminance vision at 1.44 (±0.08), and at mid spatial frequencies colour binocular summation is 1.56 (±0.02), and 1.44 (±0.05) for luminance. A linear mixed model was used for our analyses due to the different numbers of subjects in each condition and subjects repeating multiple but not all conditions. A linear mixed model analysis of our data for co-oriented gratings (*ϑ* = 0) with two factors (2 (chromaticity) × 2 (spatial frequency)) revealed that there is only a significant effect of chromaticity, *F*(1, 7.63) = 9.487, *p* = 0.016, no effect of spatial frequency, *F*(1, 7.63) = 2.101, *p* = 0.187, and no interaction, *F*(1, 7.63) = 2.058, *p* = 0.191.

Using a linear mixed model with three factors (8 (orientation) × 2 (chromaticity) × 2 (spatial frequency)), we found a main effect of orientation difference, *F*(7, 14.636) = 27.471, *p* < 0.001, reflecting decreasing summation ratios over greater orientation differences. There was also a main effect of chromaticity, *F*(1, 47.695) = 31.809, *p* < 0.001, with colour vision having larger summation ratios than luminance vision. A main effect of spatial frequency, *F*(1, 47.695) = 27.445, *p* < 0.001, reflects larger summation ratios at the low spatial frequency. Additionally, there was a significant interaction of chromaticity and spatial frequency, *F*(1, 47.695) = 13.943, *p* = 0.001. While summation ratios are relatively similar for colour and luminance vision at mid spatial frequencies, they are larger for colour vision at low spatial frequencies across all orientation differences. This interaction highlights an interesting result in the data: the near uniformly larger summation ratios for colour over luminance at low spatial frequencies. Interactions between orientation difference and chromaticity and/or spatial frequency were all non-significant, *p* > 0.05. Therefore, the increase in summation ratios for low spatial frequency colour vision does not statistically vary over orientation difference.

### Model with orientation-tuned filters

**Figure 2:**
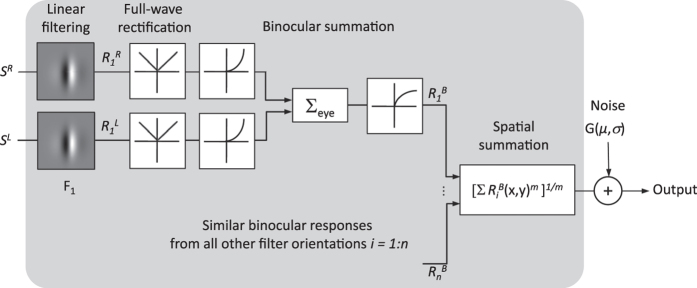
Model schematic. Model outputs are determined for monocular and dichoptic stimuli to calculate the summation ratio.

In this section we developed a model to identify orientation bandwidths of the neural detectors underlying the tuning curves in each of the four experimental conditions. In Gheiratmand & Mullen^8^, we developed a model to find the orientation bandwidths of detectors involved in monocular vision. Here, we modified the model to include a binocular summation stage as illustrated in a schematic in Fig 2.

The model involves a log Gabor filter bank representing spatial frequency- and orientation-tuned binocular detectors of the visual cortex. The purpose of the modeling is to identify the orientation bandwidth of the neural detectors (filters). Filters are Cartesian-separable log Gabors centered at the same spatial frequency as the stimulus and oriented at 0 to 170 degs in steps of 10 degs (*n* = *18*). The spatial frequency bandwidths of the filters are 2.2 and 1.6 octaves for filters tuned to 0.375 c/deg and 1.5 c/deg respectively.

The response of the *i*th binocular filter, *F*_*i*_, was found according to a nonlinear summation across monocular responses^7^,^13^,^15^,^17^,^18^:


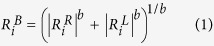


where 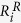 and 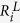 denote responses of the *i*th linear filter, *F*_*i*_, to the stimulus presented to the right and left eye respectively:


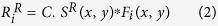



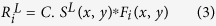


(* denotes 2-dimensional convolution.) Both stimulus and filters are 2-dimensional images in space. *C* is the contrast of the component grating, and *S*^*R*^*(x, y)* and *S*^*L*^*(x, y)* are the stimulus spatial patterns (i.e. gratings) in the right and left eye respectively.

The order of nonlinearity, *b*, in equation 1 is found based on the average binocular summation ratio (*SR*_*Bin*_) between two co-oriented vertical gratings, according to the equation *SR*_*Bin*_ = *2*^*1/b*^ 7,15,16,19. The relationship between *SR*_*Bin*_and *b* can be extracted from equations 1, 2, 3 and 5:  2.C ^*^_bin_^*b*^ = C ^*^_mon_ . *b*  is found for each stimulus condition, separately. For example, *b* equals 1.24 and 1.56 for the average chromatic stimuli at 0.375 c/deg and 1.5 c/deg, with *SR*_*Bin*_ = 1.75 and 1.56 respectively.

The model response, *R*, is calculated according to a Minkowski summation rule with order *m* (Eq. 4):


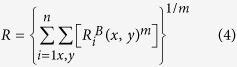


The pooling of binocular responses, 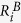, occurs across space and orientation as denoted by two summation operators in equation 4. In general, the larger the *m* value, the weaker is the summation across tuned filter responses. We chose *m* = *6* as it provided the best set of fits to the data for various stimulus conditions (compared to *m* = *4*). *m* = *6* results in *SR*  = *1.12* (2^1/6^) at 90 degs, which is consistent with our data and the implementations of spatial summation in the literature^7^,^20^,^21^,^22^.

Using the model response for an individual grating (i.e. 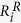 = 0 or 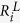 = 0 in Eq.1 for a monocular stimulus in left or right eye respectively) and dichoptic stimuli, we find *SR* as a function of *ϑ* for a range of filter orientation bandwidths (5–62 degs in steps of 1 deg). Contrasts at detection threshold are found by setting *R* equal to an arbitrary value, e.g. 1, in equation 4. All parameters in the model are fixed except the filters orientation bandwidth, which is chosen such that the RMS error between the model and the empirical orientation tuning responses is minimized. We find the optimal orientation bandwidths for individual subjects as well as the average orientation tuning curves for each experimental condition.

**Figure 3:**
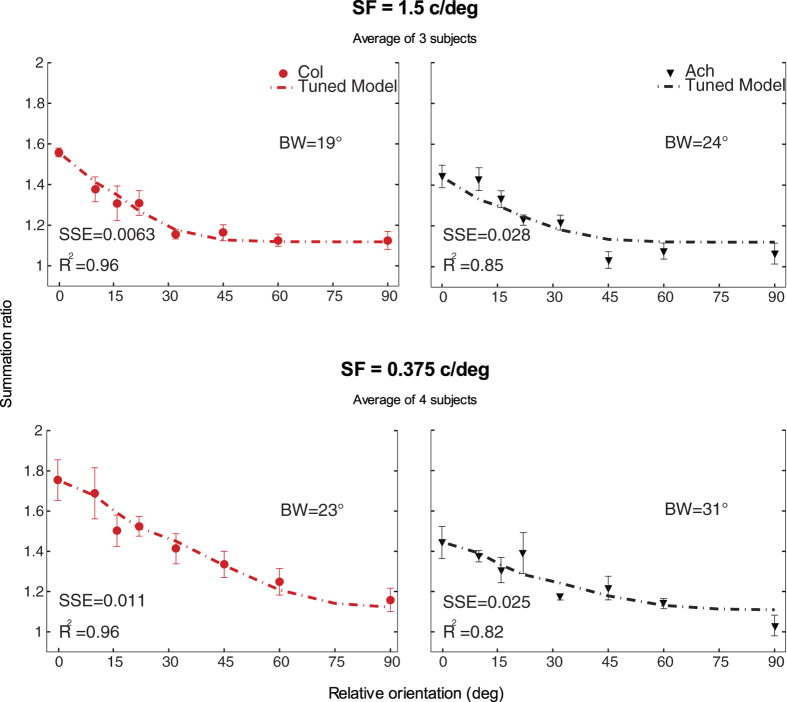
Fits of the binocular orientation-tuned model. Fits of the binocular summation model (dash-dot line) to averaged colour (red circles) and achromatic (black triangles) data at 1.5 c/deg (top panels) and 0.375 c/deg (lower panels). Orientation bandwidth estimates (HWHH in degs) and goodness of fit measures, R^2^ and SSE from the model fit are displayed on each panel. Error bars on the averaged data show the standard error of the mean (s.e.m.).

**Figure 4:**
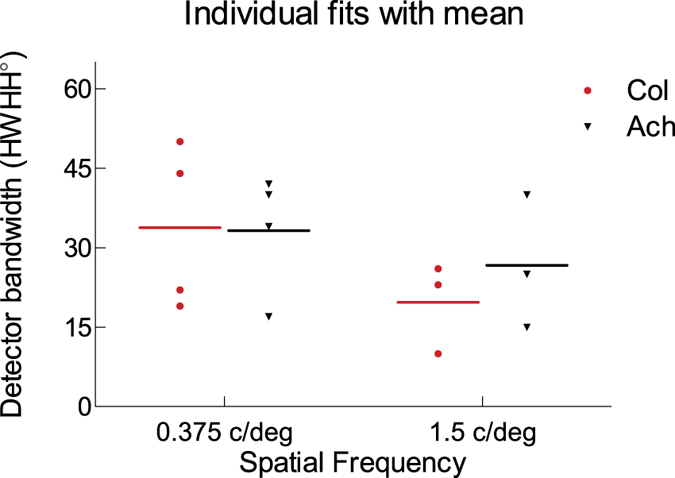
Detector bandwidth estimates (HWHH in degs) for individual data (symbols) and their average (line) for each condition. Chromatic and achromatic data are shown with red circles and black triangles, respectively.

Figure 3 shows the model fits to the averaged data for colour (red circles) and achromatic stimuli (black triangles) at 1.5 c/deg (top row) and 0.375 c/deg (bottom row). The model fits to the data of each subject are shown in Supplementary Fig. S1a (1.5 c/deg) and S1b (0.375 c/deg). The orientation bandwidth estimates (BW), R^2^ goodness of fit measure, and the sum of squared errors (SSE) of the fits are included at the inset of each graph. The model with oriented binocular filters provides reasonable fits to the average data for all four experimental conditions. At 1.5 c/deg, orientation bandwidth estimates for averaged chromatic and achromatic stimuli are 19 and 24 degs half-width at half height (HWHH), which are slightly narrower than BWs at 0.375 c/deg, at 23 and 31 degs for chromatic and achromatic stimuli respectively. Figure 4 illustrates the BW estimates for individual subjects and their average for each experiment condition. A linear mixed model test of individual orientation bandwidths with two factors (2 (chromaticity) × 2 (spatial frequency)) reveals that there are no main effects of chromaticity, *F*(1, 8.771) = 0.279, *p* = 0.611, or spatial frequency, *F*(1, 8.771) = 2.633, *p* = 0.140. Additionally, there is no significant interaction of chromaticity × spatial frequency, *F*(1, 8.771) = 0.366, *p* = 0.560. It is important to note, however that with low subject numbers, this non-significant result may be due to low statistical power. We also note that the bandwidth estimates are applicable to vertical stimuli and might vary with respect to horizontal stimuli which have higher binocular summation (Simmons & Kingdom, 1998) and also less dependence on precise binocular fusion.

As described earlier, summation ratios for the low spatial frequency chromatic stimuli are higher at all orientations compared to the other three conditions (Fig. 1). The fit of the oriented filter model is consistent with the chromatic data, however, in the next section we test whether its greater levels of summation can be explained by (binocular) isotropic detectors.

Stimuli at both spatial frequencies had identical spatial extension. Presenting stimuli with different spatial frequencies at the same size results in different orientation bandwidths, as the low spatial frequency stimulus will have fewer cycles than the middle spatial frequency, resulting in a smaller elongation to width ratio. Stimulus orientation bandwidth had no effect on monocular summation ratios in a previous study^7^. However, this property is accounted for in the model through the two-dimensional stimulus presentation and filtering.

### Model with isotropic filters

Using a model for monocular summation, Gheiratmand & Mullen^8^ found that isotropic detectors were consistent with the high levels of summation between low spatial frequency chromatic gratings of different orientations. Here, we used the binocular model described above to test whether isotropic binocular detectors can explain the higher levels of binocular summation for the low spatial frequency chromatic stimuli. We replaced the orientation-tuned filters with isotropic filters with no orientation (or spatial frequency) tuning. The red and black dotted lines in Supplementary Fig. S2 show the model response with binocular isotropic filters for *m* = *6*. With binocular isotropic detectors, the model is a very poor fit (R^2^ = −0.69) and predicts levels of summation that are consistently higher than those observed in the data. The isotropic model prediction with *m* = *3*, the *m* value that provided the best fit to the monocular colour low spatial frequency data^8^, is also shown in blue and grey lines on Supplementary Fig. S2. Additionally, we found the best isotropic model fit with a free *m* parameter to find the best Minkowski exponent fit as well. This model still did not provide a good fit for the data, R^2^ < 0. We conclude that the model does not support a hypothesis of binocular isotropic detectors as the neural mechanisms underlying the low spatial frequency chromatic orientation tuning curves.

## Discussion

Using the psychophysical method of subthreshold summation, we have found orientation-tuned responses for binocular combination for colour and achromatic vision at low and medium spatial frequencies. The underlying orientation bandwidths of the binocular mechanisms were estimated using an orientation-tuned model and were not significantly different between achromatic and chromatic contrast stimuli, or between low and mid spatial frequencies, yielding an overall average of 29 (±3, s.e.m.) degrees (HWHH) across all conditions and subjects. It is possible that any significant differences between estimated bandwidths were obscured due to low statistical power. However, our results clearly demonstrate that the binocular combination of colour contrast is orientation tuned and similar to the binocular combination of achromatic contrast at equivalent spatial frequencies. Dichoptic orientation tuning has been measured before in masking studies^23^,^24^ but often fail to account for the suppressive effects of contrast gain control. Baker & Meese^25^ accounted for the effects of orientation suppression and found a bandwidth of 8 deg for the summation stage in a supra-threshold masking paradigm with luminance stimuli of 1 c/deg. However, this narrow bandwidth may be underestimated since the effects of spatial probability summation are not taken into account, and as the authors also point out. To our knowledge, ours is the first study of the orientation tuning of binocular combination using *subthreshold* summation for either achromatic or chromatic contrast, apart from a preliminary report conducted with a method of adjustment that found a very tightly tuned orientation response for achromatic stimuli (5 deg, HWHH)^26^ on only one subject. Hence, with a more thorough methodology and model, we estimate that for stimuli presented to the two eyes with differences in orientation over 29 degrees, binocular mechanisms will effectively begin to fail whether for chromatic or achromatic contrast. Presumably, when such large orientation differences occur between the eyes, binocular neurons start to act monocularly, responding to the preferred orientation. Behaviourally, when dichoptically presented stimuli are of very different orientations, a process of binocular suppression or masking usually occurs^12^,^19^,^27^. It is interesting, however, that in our data we do not see evidence for substantial suppression, even at very large orientation differences, pointing to similar detection thresholds for stimuli presented monocularly or dichoptically. This indicates that significant binocular suppression may require higher stimulus contrasts than the near-threshold values used here, as demonstrated by threshold versus mask contrast functions for cross-oriented dichoptic masking^19^.

Our results, indicating the presence of orientation-tuned binocular responses in colour vision, are compatible with primate neurophysiological data in several ways. There is a large population of neurons in primate V1 and V2 that are sensitive to colour as well as achromatic contrast^4^,^5^, which is both spatially-tuned (peaking on average at around 1–2 cpd) and orientation-tuned (average bandwidth 30 deg (HWHH)), very close to our psychophysical estimates. Peirce *et al*.^4^ have explicitly shown that these are is binocular, with well-matched spatial properties but poorly matched colour properties between eyes. This population may potentially support our psychophysical results for either achromatic or chromatic contrast. It is interesting that our results failed to find evidence for isotropic binocular responses. Both Johnson *et al*.^5^ and Peirce *et al*.^4^ reported a small population of colour selective neurons in V1 and V2, insensitive to achromatic contrast, that are spatially lowpass and isotropic, and Peirce *et al*.^4^ also reported that they are binocular with well-matched colour properties but poorly matched spatial properties between eyes. Thus even though isotropic binocular neurons exist at the early cortical level, and are tuned to low spatial frequencies, we did not find any evidence for them psychophysically. When we replaced the oriented binocular filters in our model with isotropic binocular detectors, relatively flat functions for summation vs. orientation difference were predicted, very different from the orientation-tuned shape of the actual data (Supplementary Fig. S2). These results, however, do not exclude the presence of isotropic binocular colour responses. Psychophysical measurements based on detection thresholds reveal the most sensitive physiological responses, and potentially exclude the responses of other less sensitive populations of cortical neurons. It is possible that under other conditions, an isotropic binocular response might be revealed. Moreover, although our model does not support the presence of binocular isotropic detectors, we cannot exclude the possibility that some other model structure may be plausible that is compatible with the presence of isotropic binocular detectors.

Our results for dichoptically presented stimuli contrast with previous studies that examined subthreshold summation across different orientations under monocular viewing conditions^7^,^8^. At low spatial frequencies, there was evidence for isotropic colour responses that switched to orientation-tuned responses at higher spatial frequencies. In this study and in an earlier report of cross-orientation summation^7^, isotropic responses seem to disappear under dichoptic viewing, when binocular summation between eyes is required. It is possible that monocular isotropic pathway co-exists with a binocular one (see Figure 5 of Gheiratmand *et al*.^7^). If binocular orientation-tuned responses are more sensitive than isotropic monocular responses, it may determine threshold once two eyes are recruited. There is some evidence that binocular colour mechanisms might inhibit monocular ones, which may explain why the isotropic monocular response is only seen in monocular presentation^28^. Indeed a monocular isotropic detector could be boosting summation only to be inhibited by binocular suppression, rendering both difficult to detect in final summation levels. Ultimately, our study and model was not designed to measure suppression and therefore remains agnostic to its effects on binocular summation in threshold color vision.

There is psychophysical precedence for purely monocular low spatial frequency isotropic mechanisms. Achromatic high temporal frequency low spatial frequency stimuli are also detected through an isotropic channel^29^ and orientation tuning is acquired when stimuli are dichoptically or binocularly viewed^30^. As discussed above, while an isotropic response may be the most sensitive pathway for monocular mechanisms, binocular vision may afford greater sensitivity through orientation-tuned channels. While the isotropic monocular pathways are still present, they are not alone, and tuned channels appear to dominate binocular summation mechanisms.

Interestingly, we observed that, specifically for the chromatic stimuli at low spatial frequencies, there is significantly higher binocular summation across all orientations (Fig. 1). Higher binocular summation ratios for colour vision have also been found by Simmons & Kingdom^11^ for co-oriented stimuli, and Simmons^10^ proposed that this was due to a more linear combination of colour than achromatic contrast. In our study, elevated summation due to a more linear colour response are represented by the *b* parameter (Equation 1), which is calculated empirically by the summation level for the stimuli at zero orientation difference. There is a proportional inverse relationship between psychometric slope and binocular summation, with shallower slopes predicting a more linear response and greater summation^19^,^31^. However our slopes could not be estimated sufficiently accurately to allow this to be tested.

Colour responses may be more linear due to a lack of sub-cortical contrast normalization in P cells^32^ and/or a more linear combination at the binocular neuron. In the mouse (which doesn’t have trichromatic colour vision), it has been shown that physiologically, binocular neurons combine signals differently depending on the type of signal input. Longordo *et al*.^33^ demonstrated that binocular neurons combine monocular signals using sublinear integration when they have higher response levels, such as to preferred orientations, binocular disparities, or higher contrasts. However if the incoming signals are weaker, binocular integration is increasingly linear. Weaker linearly combined monocular signals were estimated to raise action potential firing rates across *all* orientations resulting in a response curve with the same orientation tuning bandwidth as from sublinear integration, but a less distinctive preference for a particular orientation. This effect is similar to the observed raising of low spatial frequency binocular colour summation ratios across orientation. It is plausible that a low spatial frequency colour stimulus is a ‘weaker’ input for many binocular neurons leading to a more linear summation at the binocular neuron, as supported by the more linear *b* parameter calculated from our data.

While the mechanisms underlying increased summation in binocular low spatial frequency colour vision are presently unknown, we have provided clear evidence for orientation tuning. Whereas monocular low spatial frequency colour contrast detection may be isotropic or very broadly tuned, acting as a blob detector and for chromatic filling in, binocular colour vision appears well capable of oriented tasks such as edge detection and shape processing. These results suggest that any untuned monocular signal acquires orientation tuning with further processing through binocular mechanisms.

## Methods

### Apparatus

Stimuli were produced by a 14-bit contrast resolution ViSaGe video-graphics card (Cambridge Research Systems, Kent, UK). They were viewed with a custom-built, modified 8-mirror Wheatstone stereoscope (as used in Gheiratmand *et al*.^7^,^8^) with stimuli displayed on the right and left side of the monitor and then directed to the right and left eye respectively. Binocular fusion was achieved by optimizing the stimulus position on the screen, and a separate preliminary experiment asked subjects to align two vertical lines viewed through the stereoscope. The experiment was repeated 10 times and the averaged location was used in the main experiment. These test results were stable for each subject and there was no reported difficulty in binocular fusion. The monitor used for all testing was an Iiyama Vision Master Pro 513 CRT monitor (Iiyama Corporation) (1024 × 768 resolution, 120 Hz refresh rate, 43 cd/m^2^ mean luminance, and viewing distance of 58 cm). Gamma correction and colour calibration of this monitor are described in Gheiratmand & Mullen^8^.

### Observers

There were 7 participants in this study. However not all participants completed all experiment conditions; there were 3 subjects for each middle spatial frequency condition and 4 for each low spatial frequency condition. Two are authors (AC and MG) while the remaining five (AB, IO, MS, RSE, and SK) were naive as to the purpose of the experiment. All participants had normal or corrected-to-normal visual acuity and normal colour vision, as assessed with the Farnsworth-Munsell 100 Hue test. The experiments were performed in accordance with the Declaration of Helsinki and approved by the institutional ethics committee of the Research Institute of McGill University Health Centre. All participants signed an information consent form.

### Colour space

Stimuli are characterized by a 3-dimensional cone-contrast space in which each axis is the stimulus contrast for one of the three cone types. Details on the derivation of this cone-contrast space can be found in Gheiratmand & Mullen^8^. Red-green isoluminance points for each participant were measured at each spatial frequency using a minimum motion task as described in Gheiratmand & Mullen^8^.

### Stimuli

Test stimuli were chromatic or achromatic sine-wave gratings (phase = 0) of 0.375 or 1.5 c/deg displayed within a circular patch (diameter of 10 deg) with the edges smoothed using a raised-cosine envelope. Stimuli were presented in a temporal Gaussian envelope (σ = 125 ms). Different gratings were presented on alternate monitor frames. Grating stimuli were either presented monocularly alone to the right or left eye, or dichoptically combined as pairs. During monocular viewing, the other eye viewed the unmodulated, mean luminance. Examples of the stimuli and their combinations can be found in Gheiratmand & Mullen^8^. The stimulus orientation was varied relative to the vertical axis and the orientation difference is defined by the difference in orientation between the dichoptic pair. There were 8 orientation differences (0, 10, 16, 22.5, 32, 45, 60, 90), so that, as example, a dichoptic pair of orientation difference 10 deg would consist of a pair of gratings at +5 deg, right oblique, and −5 deg, left oblique, presented to the right and left eye, respectively. There was no fixation point present during the experiment.

### Protocols and analysis

We used the psychophysical method of subthreshold summation to measure summation between responses to two oriented gratings presented to separate eyes^21^,^30^,^34^,^35^,^36^. Here we measure the reduction in contrast detection thresholds for two stimuli presented dichoptically over one component stimulus presented alone. Detection thresholds were obtained for left oblique gratings, right oblique gratings, and dichoptic pairs for each orientation difference in all conditions (Chromaticity (2) × SF (2) × orientation (8)). Participants completed this experiment over multiple sittings and with a pseudo-randomized balanced block design. They completed one of the four spatial frequency and chromaticity conditions before moving to the next, in random order. Stimuli were blocked both in terms of orientation and presentation (plaid, left oblique grating, or right oblique grating) with each block containing 20 trials of six separate contrast levels of one stimulus. Participants were told which stimulus orientation and presentation was to be presented before each block. Blocks took approximately six minutes to complete and were followed by rest periods. Detection thresholds were obtained using a 2 Alternative Forced Choice (2AFC) method of constant stimuli, in which one interval contained mean luminance and the other contained a stimulus. Each stimulus interval was 500 ms, with an inter-stimulus interval of 400 ms. Participants indicated which interval contained a stimulus and were given auditory feedback. Detection thresholds were determined from the fit of the psychometric functions of percent correct per stimulus contrast level, with 100–140 trials per level. A Weibull function with the lapse rate constrained to 0.02 was fitted to the psychometric data using the *psignifit* toolbox for Matlab 7.2^37^. Detection threshold corresponded to 81.6% correct detection. We did not find significant differences in detection threshold between oblique gratings presented to the right or left eyes, or between monocular gratings presented at different orientations. These data were combined so that there were 300–660 trials per contrast level and the two component gratings had identical contrasts at detection threshold.

Summation Ratios (SR) are computed according to Eq. 5:


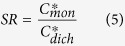


where C * represents grating contrast at detection threshold, 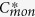 denotes the contrast at detection threshold of a single component grating presented monocularly, and 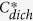 the contrast of either component grating in a dichoptic pair at detection threshold. SRs usually vary between 1 and 2, where SR = 1 denotes a lack of summation and SR = 2 indicates a linear neural response summation between the two eyes; 1 < SR < 2 indicates weaker forms of inter-ocular summation^10^,^11^. Derivation of summation ratios is described in more detail in Gheiratmand & Mullen^8^. To find the binocular orientation tuning curves, we measured summation ratio as a function of the relative orientation between the two gratings (*ϑ*) presented in a dichoptic pair. In order to derive from these data estimates of the orientation bandwidths of the neural detectors underlying the psychophysical responses we employ a model described in the Results section.

## Additional Information

**How to cite this article**: Gheiratmand, M. *et al*. Orientation tuning of binocular summation: a comparison of colour to achromatic contrast. *Sci. Rep*. **6**, 25692; doi: 10.1038/srep25692 (2016).

## Supplementary Material

Supplementary Information
