# Supplementary material for: Orientation tuning of binocular summation: a comparison of colour to achromatic contrast
Source: Sci Rep. 2016 May 11;6:25692. doi: 10.1038/srep25692 (PMC4863369; doi:10.1038/srep25692)
Supplement: Supplementary Information [file srep25692-s1.doc]

**Orientation tuning of binocular summation: a comparison of colour to achromatic contrast**

Mina Gheiratmand, Avital Cherniawsky, & Kathy T. Mullen

*McGill Vision Research, Dept. of Ophthalmology, McGill University, Montreal, Canada*

**Supplementary Figures and Legends**

Figure S1 Orientation tuned model fits to individual subjects

Figure S2 Isotropic model fits to average data


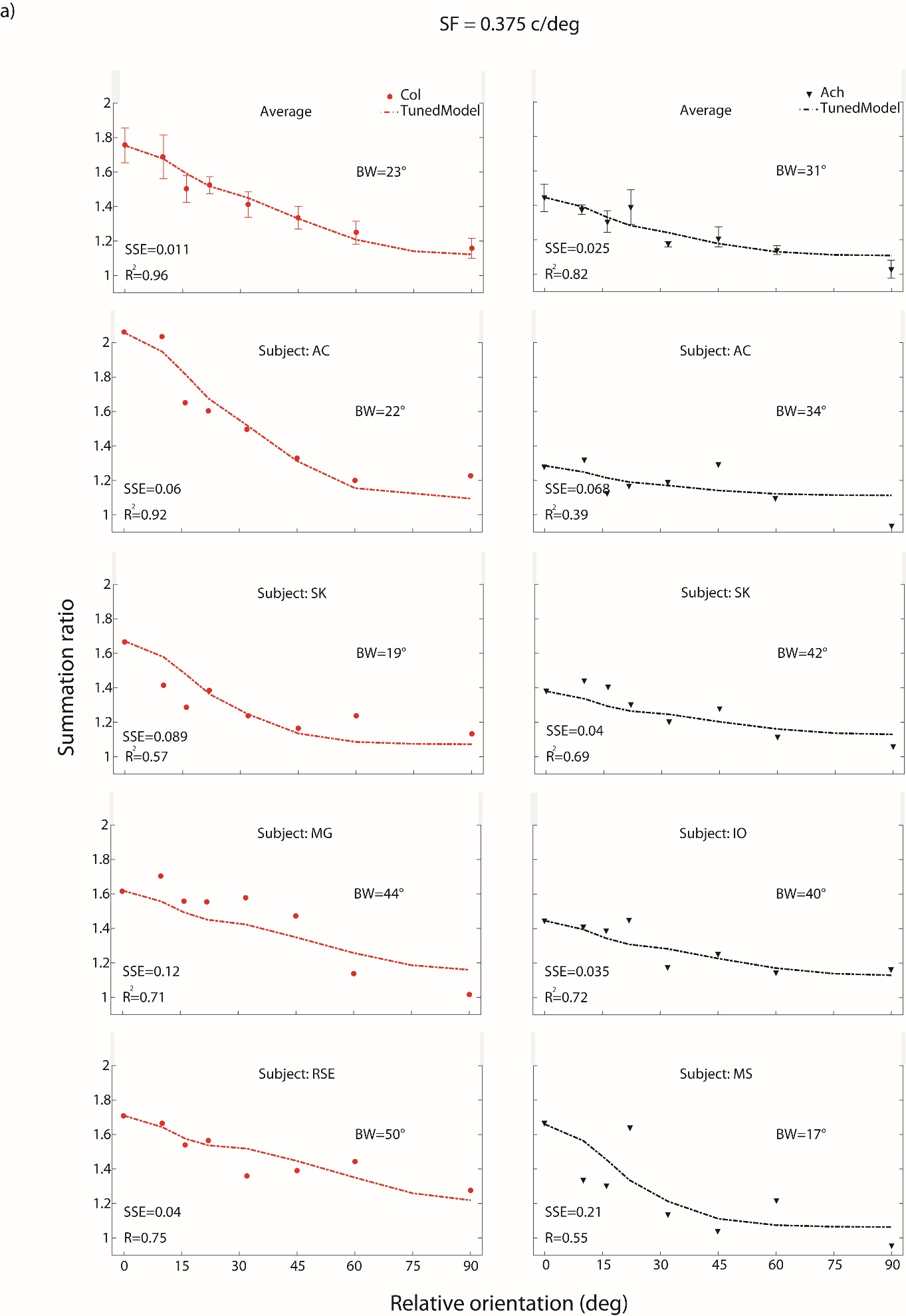


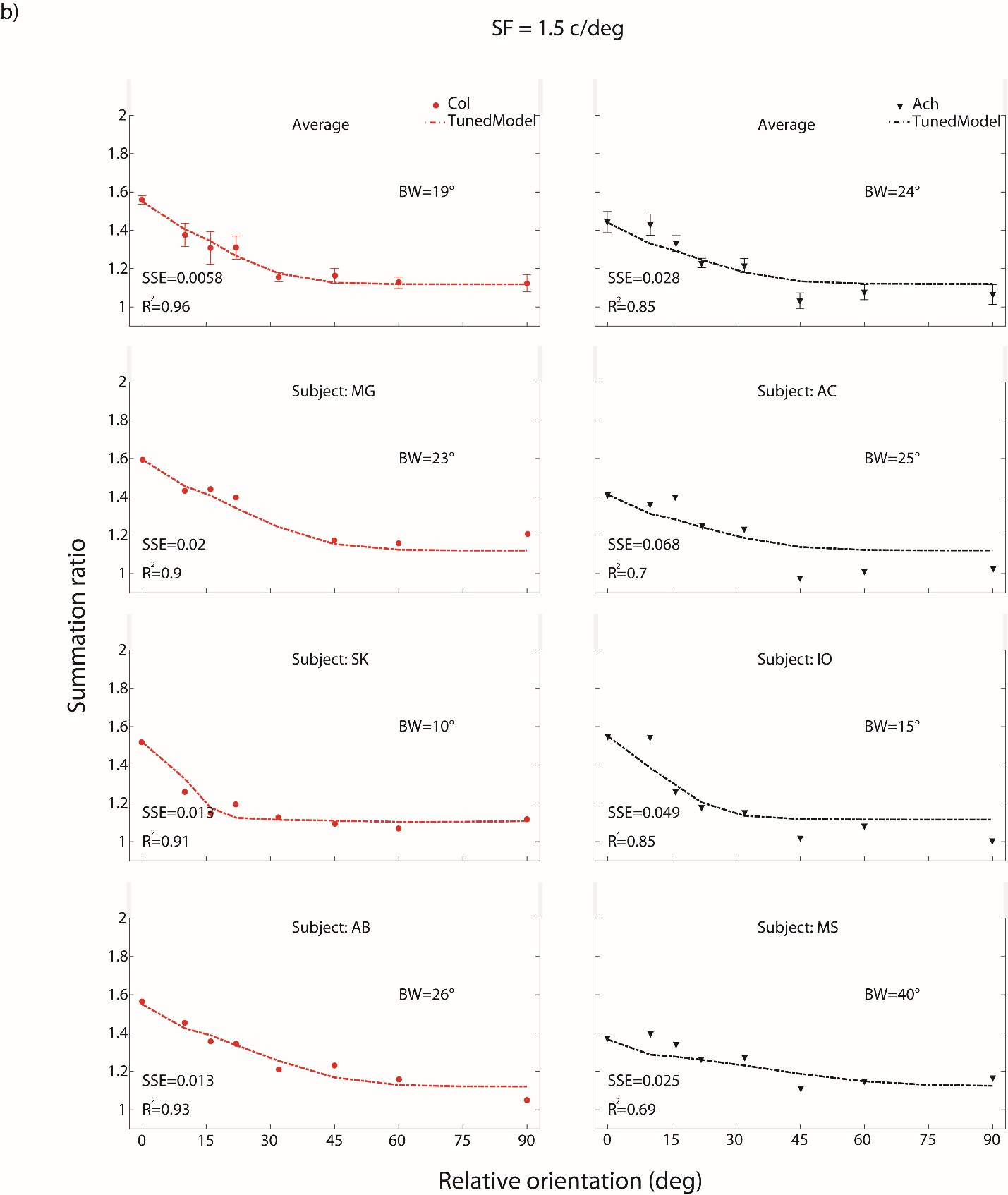


Figure S1. Model fits for average (with standard error of the mean) and individual subject orientation tuning data for color (red circles) and achromatic (black triangles) at the 0.375 c/deg (**a**) and the 1.5 c/deg (**b**). The neural detector bandwidth estimates (BW) and goodness of fit measures, R^2^ and SSE are displayed on each graph. Summation ratios are plotted against the relative orientation difference of the component stimuli. Standard deviation for individual summation ratio points, based on Gaussian error propagation, is on average 0.08.


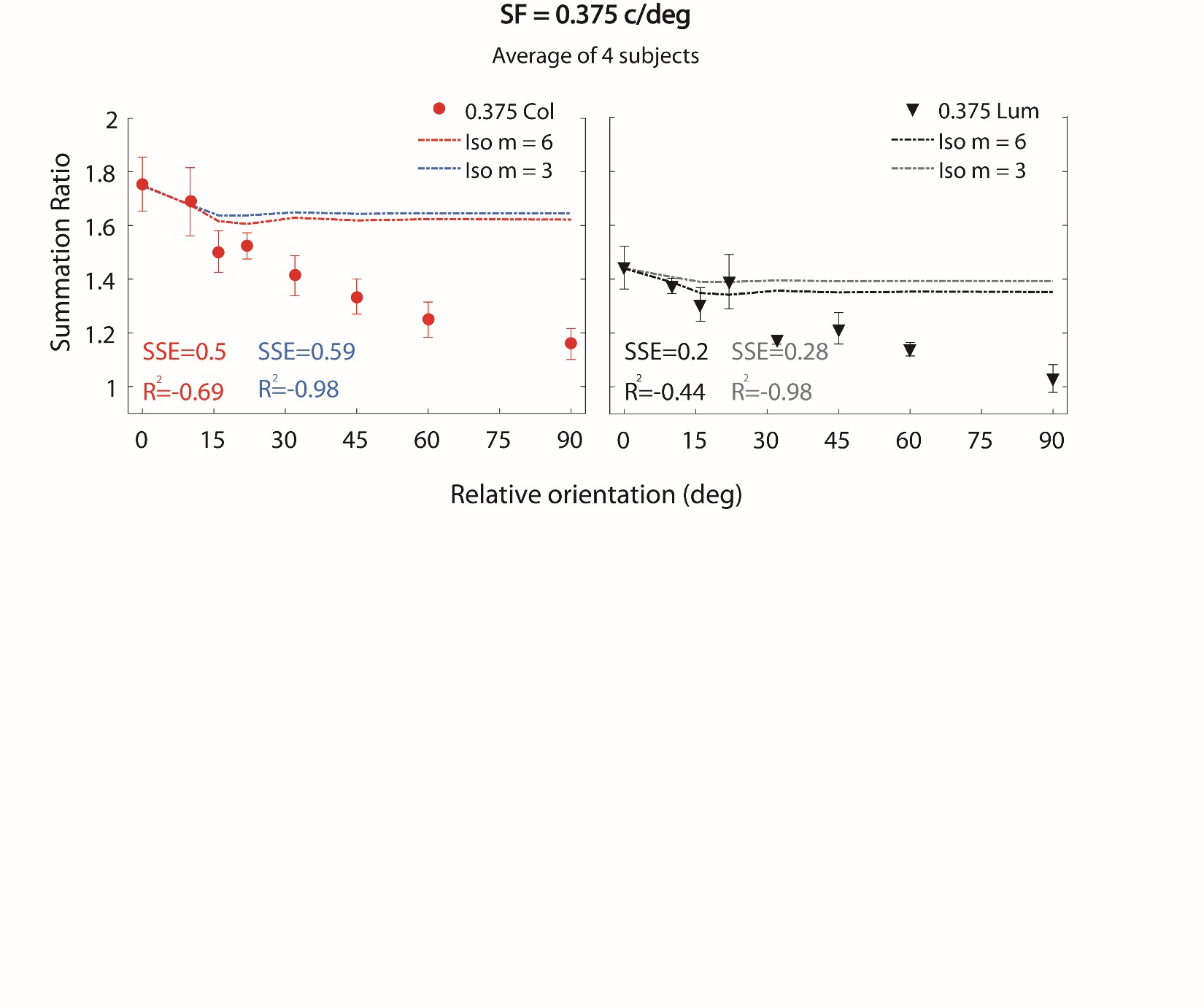


Figure S2. Isotropic model fits to the average (with standard error of the mean) color (left) and achromatic (right) data for the low spatial frequency condition. Goodness of fit measures, R^2^ and SSE are displayed on each graph. Model outputs with a Minkowski exponent (m) of 6 are shown in red for color and black for achromatic. Model outputs with an m of 3 are shown in blue for color and grey for achromatic. Summation ratios are plotted against the relative orientation difference of the component. Note that R^2^ is negative for all fits.
